# Implications of designing a bromelain loaded enteric nanoformulation on its stability and anti-inflammatory potential upon oral administration

**DOI:** 10.1039/c7ra13555f

**Published:** 2018-01-11

**Authors:** Manu Sharma, Rishu Sharma

**Affiliations:** Department of Pharmacy, Banasthali Vidyapith Banasthali Rajasthan India-304022 sharmamanu10@gmail.com +91-9694881221

## Abstract

The objective of the present investigation was to develop an enteric nano-formulation of bromelain to improve its stability and anti-inflammatory potential. Bromelain loaded nanoparticles (Br-NPs) were developed using a Eudragit L 100 polymer by a double emulsion solvent evaporation method to obtain gastro-resistant properties. Br-NPs were characterized for particle size (248.89 ± 22.76 nm), zeta potential (−27.34 ± 2.17 mV), entrapment efficiency (85.42 ± 5.34%), surface morphology (spherical) and *in vitro* release profile. Infrared spectroscopy confirmed the entrapment of bromelain while thermal and pXRD analysis concomitantly corroborated the reduced crystallinity of bromelain in nanoparticles. Formulations showed gastro-resistant behavior at gastric pH and sustained bromelain release up to 10 h in phosphate buffer at pH 6.8 and followed Higuchi square root release kinetics. The optimized lyophilized formulation ensured 2 year shelf-life at room temperature. *In vivo* studies revealed significantly improved performance of entrapped bromelain in inhibiting carrageenan induced paw edema by mitigating leucocyte migration and release of nitric oxide, TNFα and IL-1β in paw compared to bromelain solution. In conclusion, enteric Br-NPs could be a viable drug delivery system for effective oral bromelain delivery in inflammatory conditions.

## Abstract 2

The objective of the present investigation was to develop an enteric nano-formulation of bromelain to improve its stability and anti-inflammatory potential.

## Graphical abstract

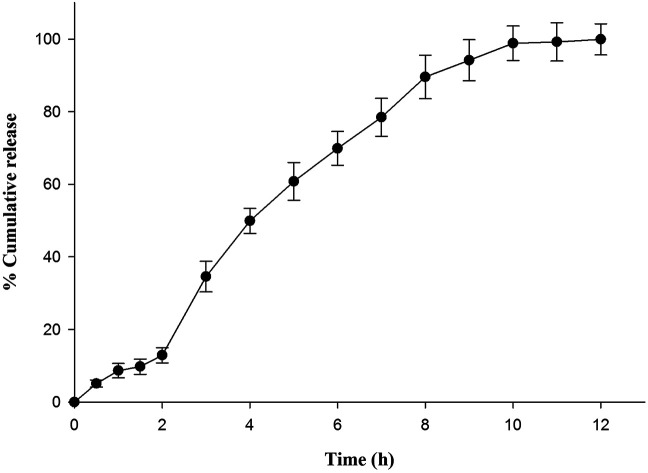

## 1. Introduction

Inflammation is an essential biological response manifested to preserve the integrity of tissues functioning in reply to cellular injury caused by physical, chemical or infective insult.^1^ However, frequent insults erroneously lead to initiation and progression of cellular inflammation to normal tissue damage by chronic activation of resident macrophages and cells adjacent to lesions and consequent overproduction of pro-inflammatory mediators.^2,3^ Excessive inflammation also serves as a prominent risk factor of various chronic pathological conditions like cancer,^4^ neurodegenerative,^5^ cardiovascular,^6^ respiratory,^7^ metabolic and autoimmune disorders.^8^ Inflammation is strongly integrated with pain which sometimes progress to physical disability, restlessness, feelings of severe despondency and dejection. Thus, treatment of inflammation becomes imperative to minimize pain and ameliorate quality of life. Conventional therapies for pain and inflammation management utilize steroidal and non-steroidal anti-inflammatory drugs (NSAIDS). However, perennial utilization of the above mentioned drugs has frequent and potential hazardous side effects, low efficacy and poor patient compliance.^9–11^ Development of effective and safe treatment of inflammation is of high clinical priority.^12^

In recent years, a variety of dietary compounds derived from natural sources have been widely consumed for minor and major illness by almost 80% of world population.^13^ Several epidemiological studies have indicated beneficial effect of commonly consumed dietary fruits and vegetables over human health in inflammatory conditions.^14^ Among dietary compounds, bromelain (sulfhydryl proteolytic enzymes) obtained from stem of pine apple (*Ananas comosus*) is gaining enormous attention due to its higher safety and efficacy index at lower cost.^15,16^ It exhibits anti-inflammatory potential in several clinical conditions involving acute and chronic inflammation like infection,^17^ osteoarthritis,^18^ colonic inflammation^19,20^ and cancer.^21^ Bromelain detaches particular cell surface molecules associated with lymphocyte and neutrophil migration to the site of inflammation and specifically removes CD28 chemokine receptor for anti-inflammatory activity.^22–25^ Bromelain also significantly blocked activation and production of cytokines and inflammatory mediators in cultured leukocytes or human intestinal cells.^26,27^ Thus, outcomes of various *in vitro* investigations demonstrated that bromelain can be a favorable prospect for establishing future oral enzyme remedies for patients with acute and chronic inflammatory conditions.

However, clinical usage of bromelain *via* oral route is so far a challenge due to its high propensity of degradation and instability in acidic environment of stomach.^23,28^ Being proteineous in nature, bromelain's denaturation and/or aggregation may also lead to loss of its therapeutic efficacy along with unpredictable immunogenic and toxic side effects.^28,29^ Therefore, endeavors are needed to develop suitable pH sensitive novel drug delivery system for bromelain which can bypass harsh conditions of stomach and deliver bromelain efficiently at therapeutic dose.

Polymeric nanoparticulate formulations are gaining much attention nowadays for controlled delivery of biomolecules.^30^ Small size of nano-formulations facilitates rapid gastric emptying, reproducible transit through GIT and target specific delivery. They also offer additional advantages of protecting encapsulated drug, controlled drug release, reduced toxicity and immunogenic potential of proteins over conventional system.^30–32^ However, success of proteins as pharmaceuticals depends on development of site specific drug delivery system by selecting a suitable polymer that allow protein to acquire stability and access to target site for proper duration.^33^ pH sensitive enteric polymers like hydroxypropyl methyl cellulose phthalate (HPMCP), cellulose acetate phthalate (CAP) and Eudragit® polymers are widely used to coat powders, tablets and capsules in pharmaceutical industries. HPMCP and CAP are enormously utilized for preparing gastro-resistant oral formulations of drugs. However, protein delivery is susceptible to deterioration due to solubility of HPMCP at pH 5.0–5.5 facilitating burst release and acidic environment generated by CAP.^34,35^ Therefore, Eudragit® polymers like Eudragit L 100 and Eudragit S 100 are rapidly gaining attention in recent years for delivery of bio-molecules *via* oral route. They offer advantages of pH-dependent release profiles, resistance to acidic environment of stomach, higher drug loading, sustained/controlled release behavior and a high level of stability.^36–38^

Henceforth, in the background of above facts objectives of current study was to design and optimize enteric nanoparticulate formulation of bromelain using Eudragit L 100 as a polymer. Furthermore, optimized formulation was characterized for its release behavior, stability and pharmacodynamic response.

## 2. Results and discussion

### 2.1 Formulation and optimization of Br-NPs

The effect of drug–polymer ratio, homogenization speed, volume of external phase, type and concentration of surfactant on bromelain loading, particle size and zeta potential of various batches formulated by double emulsion solvent evaporation method are represented in Table 1. The entrapment efficiency and particle size increased on varying drug–polymer ratio from 1 : 5 to 1 : 15. The relative increase in amount of polymer in polymeric solution facilitates faster film formation across the core material due to shorter time required for gelation of polymeric composition. The quick solidification of polymeric membrane fixed the structure of particles leading to slowed counter diffusion of solvent. Thus, higher polymer loads reduced drug leaching rate from dispersed to continuous phase.^39^ However, no substantially significant improvement in entrapment efficiency was observed for batch (L_4_) prepared with drug polymer ratio 1 : 20 compared to 1 : 15 (L_3_). This might be contributed by reduced actual drug loading. Drug–polymer ratio also exhibited a significant influence on PDI due to its direct effect on thickness of polymer coating. Polydispersity index, an indicator of particle size distribution of various batches varied from 0.213–0.412. PDI of batches in the range of 0.15 to 0.30 indicated degree of homogeneity of size distribution of nanoparticles while greater than 0.30 indicated heterogeneity of dispersion. The increased viscosity of organic phase with relative increment of polymer load had resulted in genesis of coarse dispersion due to deficiency of ample energy to conquer viscous forces (Table 1).^39,40^

**Table 1:** Effect of formulation variables on entrapment efficiency, particle size and zeta potential

Formulation code	Drug : polymer ratio	Homogenization speed (rpm)	External aqueous phase volume (ml)	Surfactant		Particle size (nm ± SD)	Zeta potential (mV ± SD)	Poly-dispersity index (PDI ± SD)	Entrapment efficiency (% ± SD)	Viscosity of organic phase (cps ± SD)
				Tween-80 (% w/v)	PF-68 (% w/v)					
L_1_	1 : 5	12 500	25	0.1	—	153.45 ± 17.42	−19.43 ± 3.46	0.42 ± 0.01	56.43 ± 6.02	0.471 ± 0.02
L_2_	1 : 10	12 500	25	0.1	—	215.25 ± 20.43	−25.46 ± 4.15	0.36 ± 0.03	70.46 ± 5.41	0.501 ± 0.03
L_3_	1 : 15	12 500	25	0.1	—	248.89 ± 22.76	−27.34 ± 2.17	0.23 ± 0.01	85.42 ± 5.34	0.528 ± 0.02
L_4_	1 : 20	12 500	25	0.1	—	365.56 ± 21.43	−28.01 ± 2.87	0.39 ± 0.04	79.78 ± 4.91	0.576 ± 0.01
L_5_	1 : 15	12 500	25	—	0.1	314.54 ± 18.21	−25.04 ± 3.14	0.41 ± 0.03	77.98 ± 10.12	0.527 ± 0.02
L_6_	1 : 15	12 500	25	0.05	—	345.45 ± 23.12	−27.10 ± 3.87	0.46 ± 0.02	78.23 ± 12.31	0.527 ± 0.02
L_7_	1 : 15	12 500	25	0.2	—	212.59 ± 22.87	−24.89 ± 3.13	0.29 ± 0.03	75.24 ± 10.85	0.526 ± 0.03
L_8_	1 : 15	10 000	25	0.1	—	475.23 ± 25.23	−25.12 ± 2.45	0.62 ± 0.03	76.12 ± 10.56	0.525 ± 0.02
L_9_	1 : 15	15 000	25	0.1	—	320.36 ± 28.03	−24.59 ± 3.01	0.35 ± 0.04	69.23 ± 12.01	0.524 ± 0.03
L_10_	1 : 15	12 500	50	0.1	—	314.27 ± 21.34	−24.06 ± 3.12	0.48 ± 0.02	77.23 ± 8.95	0.527 ± 0.02

Increase in homogenization speed showed significant impact on reduction of particle size and polydispersity index whereas enhanced entrapment efficiency (Table 1). This might be attributed to the formation of uniform micellar structures. On the contrary, further increment of homogenization speed (15 000 rpm) increased particle size and reduced entrapment efficiency (Table 1). This could be due to high shear forces facilitating formation of non-uniform interfacial barrier between two phases during micelle formation leading to coalescence of dispersed phase.

Among surfactants, tween-80 showed higher encapsulation of bromelain compared pluronic F-68 at 0.1% w/v concentration. This confirmed that tween-80 formed stable primary emulsion by providing mechanical and thermodynamically stable barrier at interface preventing aggregation and coalescence of particles. Similar results had been obtained for encapsulation of hydrophilic drugs like cephalexin, beta-lactoglobulin and Brucella abortus.^41–43^ The decrease in surfactant concentration (0.05% w/v) increased particle size whereas reduced entrapment efficiency confirming the need of suitable amount of surfactant for preparing stable colloidal system. However, decreased entrapment efficiency with higher concentration of tween-80 might be attributed to solubilizing effect of surfactant. The magnitude of surface charge represented as zeta potential for various batches varied from −19.43 to −28.01 mV. Colloidal systems with zeta potential having numerical values near ±30 mV ensures their considerable physical stability.^42^

Increase in volume of external phase showed significant effect on particle size, PDI and entrapment efficiency. Particle size and PDI increased while entrapment efficiency reduced with increase of external phase volume from 25 ml to 50 ml. This might be due to deficit of sufficient shear forces for the formation of uniform stable micellar structures.^40^ Thus, depending upon the results obtained, formulation (L_3_) prepared with optimized variables like drug–polymer ratio 1 : 15, homogenization speed 12 500 rpm, tween-80 (0.1% w/v) as stabilizer and external phase volume of 25 ml was designated for further investigations.

### 2.2 Effect of pH on formulation stability

It was observed that pH of surrounding media influenced the colloidal behavior of formulation. Nanoparticles showed aggregation (size of aggregates being 341.24 ± 24.67 μm), reduced zeta potential (−0.27 ± 0.01 mV) and increased PDI (0.98 ± 0.02) suggesting reduced colloidal behavior of system under acidic environment. The protonation of carboxylic groups of methacrylic acid (*p*K_a_ ∼4.23) in acidic media (pH < *p*K_a_) increased van der Waal forces of attraction between polymeric molecules and facilitated aggregation of nanoparticles.^44^ Earlier studies had reported retention of particles greater than 500 μm in stomach until fragmented to smaller size.^33^ However, size of aggregates observed was <500 μm indicating that aggregation of nanoparticles would not interfere with gastric emptying of nanoparticles. Optimized formulation incubated in phosphate buffer pH 5.0, 6.8 and 7.4 had remained uniformly dispersed as indicated by significantly higher zeta potential in comparison to HCl buffer pH 1.2 (Table 2). Since *p*K_a_ < pH, polymer remained protonated and electrostatic repulsive forces maintained the uniform dispersibility of nanoparticles at pH conditions similar to intestine. The numerical values of zeta potential (near – 30 mV) and PDI (<0.3) of formulation at intestinal pH indicated that aggregates would undergo disaggregation during passage from stomach to intestine resulting in increased effective surface area.^44,45^

**Table 2:** Effect of surrounding pH on particle size, zeta potential and drug release behavior of formulation after incubation of Br-NPs at respective pH for 2 h

S. No.	pH	Particle size (nm ± S.D.)	Zeta potential (mV)	PDI	Drug release (%)
1	5.0	428.19 ± 32.16	−15.34 ± 2.17	0.65 ± 0.01	18.23 ± 3.25
2	6.8	258.81 ± 31.06	−25.34 ± 2.24	0.25 ± 0.02	32.45 ± 4.23
3	7.4	241.64 ± 22.76	−28.69 ± 3.21	0.23 ± 0.01	37.12 ± 5.01

Drug release (12.98 ± 2.45%) from optimized formulation in HCl buffer pH 1.2 indicated insufficiently coated surface of nanoparticles. The nanoparticles were only slightly swollen but remained intact and aggregated since Eudragit L 100 being insoluble at acidic pH. Thus, polymeric nanoparticles would maintain stability of bromelain during passage across stomach. The increased percentage drug release was observed with pH rise (5.0 to 7.4) due to increased ionization of polymer which prevented aggregation of nanoparticles and provided higher effective surface area for solvent contact and drug diffusion (Table 2).^46^

### 2.3 Drug release study

**Fig. 1:**
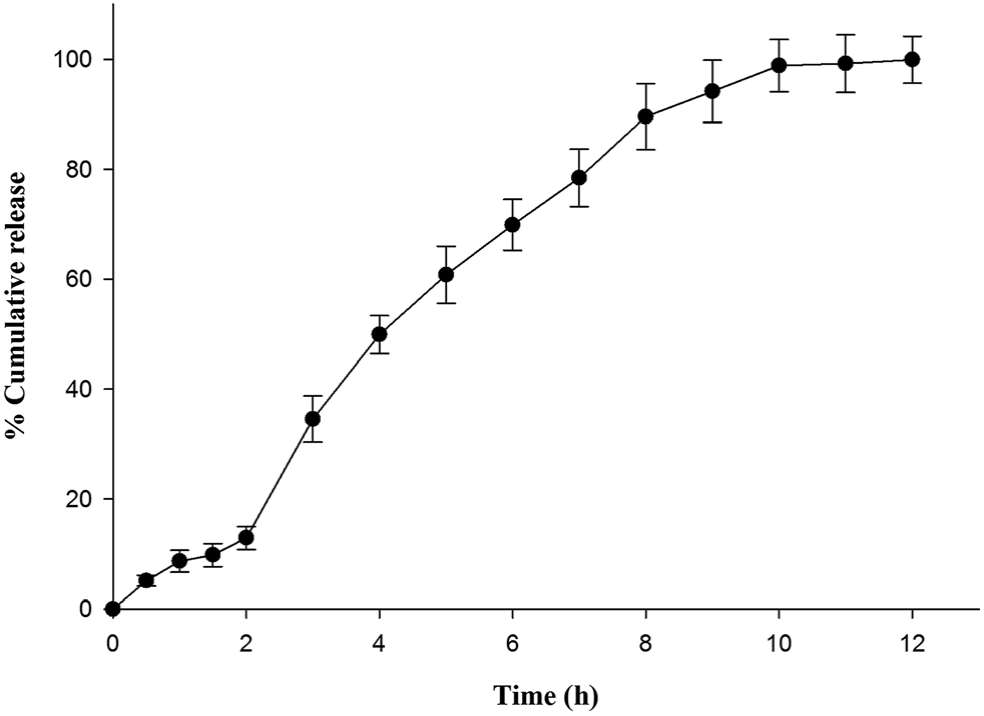
*In vitro* release behavior of optimized formulation (L_3_) in pH progressive media (HCl buffer pH 1.2 for initial 2 h followed by phosphate buffer pH 6.8).

*In vitro* drug release behavior of optimized formulation was investigated in pH progressive media in order to determine the potential of optimized formulation (L_3_) to mimic enteric and controlled release behavior. Fig. 1 illustrates almost 13% drug release in HCl buffer pH 1.2 within 2 h. Drug release in simulated acidic environment might be due to leaching of drug from insufficiently coated surfaces or film defects arising during process of lyophilization. However, nanoparticles remained intact in HCl buffer pH 1.2 due to insolubility of Eudragit L 100. Optimized formulation showed burst drug release as pH of dissolution fluid was increased to pH 6.8 by addition of disodium hydrogen orthophosphate after 2 h. The abrupt release might be contributed by quick diffusion and elution of weakly bound drug along with dissolution of outer polymeric layer. On the contrary, succeeding prolonged release for 10 h indicated slower dissolution of polymeric layer. Further analysis of release data by substituting it to different release kinetic models showed best fit to Higuchi square root release kinetic model which was confirmed by comparing *R*^2^ value obtained for different release kinetic models *i.e.* zero order (0.943), first order (0.798), Higuchi (0.973) and Korsmeyer Peppas release kinetic (0.963) model. The numerical value of release exponent (*n* = 0.694) revealed anomalous release behavior of drug *i.e.* diffusion of drug along with dissolution of polymer contributed to release.

### 2.4 ATR-FTIR spectroscopy

**Fig. 2:**
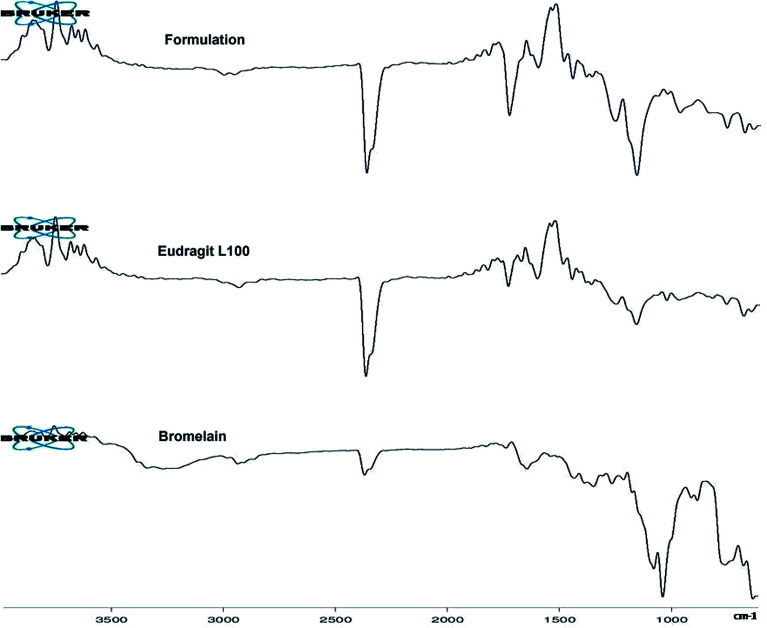
ATR-FTIR spectra of bromelain, Eudragit L 100 and optimized formulation.

Fig. 2 represents ATR-FTIR spectra of Eudragit L 100, bromelain and optimized formulation. Eudragit L 100, an anionic copolymer of methacrylic and methylmethacrylate exhibited characteristic predominant peak of C=O stretching vibration of ester group at 1723.59 cm^−1^ in its spectra. Bromelain showed characteristic peak at 3258.17 cm^−1^ (N–H stretching vibrations of secondary amide), 2926.44 cm^−1^ (aliphatic C–H stretching vibration), 2360.23 cm^−1^ (S–H stretching), 1727.75 cm^−1^ and 1634.08 cm^−1^ (C=O stretch of aldehyde and amide group), 1337.53 cm^−1^ (N–O symmetric stretch), 1423.40 cm^−1^ (C–N stretch of primary amide), 900.77 and 874.50 cm^−1^ (out of plane C–H bending of aromatic residue of tryptophan or tyrosine),749.53 cm^−1^ (out-of-plane N–H wagging of secondary amide) and between 700–600 cm^−1^ (C–S stretch of sulfides and disulfides). Peaks in the spectra of bromelain loaded nanoparticles at 3386.2 cm^−1^, 837 cm^−1^, 1153.03 cm^−1^ and 700–600 cm^−1^ for substituted secondary amide, substituted aromatic ring, C–S stretch of sulfides and disulfides suggested bromelain entrapment.

### 2.5 pXRD

**Fig. 3:**
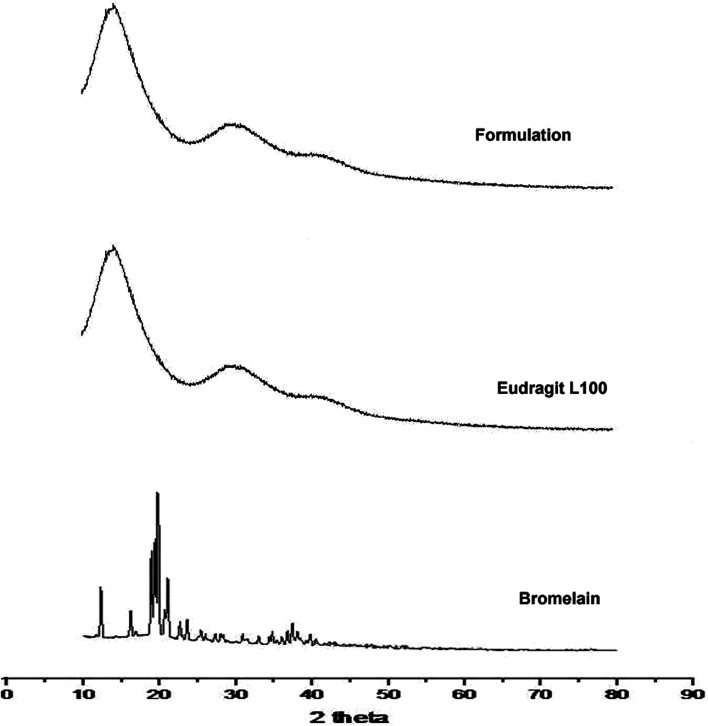
X-ray diffraction pattern of bromelain, Eudragit L 100 and optimized formulation.

Diffraction pattern of bromelain, polymer and optimized formulation is represented in Fig. 3. Eudragit L 100 diffractogram displayed diffused peaks indicating its amorphous behavior. The intense peaks at 18.72°, 19.54°, 20.34° and 21.04° were observed in the diffractogram of bromelain whereas low intensity peaks at 12.47°, 16.18° and 23.82°. This indicates semi-crystalline behavior of bromelain. However, characteristic peaks of bromelain were absent in diffractogram of formulation. This further confirmed uniform molecular dispersion of drug in amorphous form in polymeric nanoparticles.

### 2.6 DSC

**Fig. 4:**
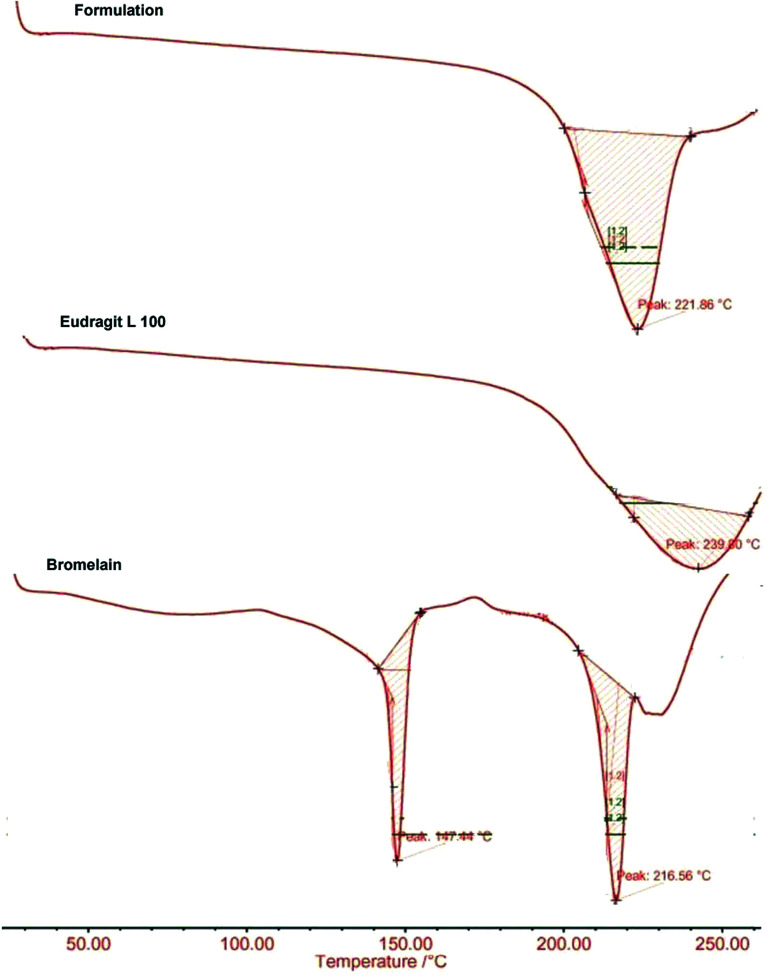
DSC thermogram of bromelain, Eudragit L 100 and optimized formulation.

DSC thermograms of bromelain, Eudragit L100 and optimized formulation are presented in Fig. 4. Eudragit L100 exhibited a broad endothermic peak at 221.86 °C with heat of fusion 0.360 J g^−1^. The sharp melting endothermic peaks at 147.44 °C and 216.56 °C with corresponding heat of fusion 7.789 J g^−1^ and 8.321 J g^−1^ respectively characterizes thermal behavior of bromelain. While thermogram of optimized formulation showed a single broad peak at 239.80 °C with heat of fusion 0.366 J g^−1^ devoid of characteristic peaks of bromelain. The results indicated reduced crystallinity of bromelain in polymeric matrix which are also supported by X-ray analysis.^47^

### 2.7 Scanning electron microscopy

**Fig. 5:**
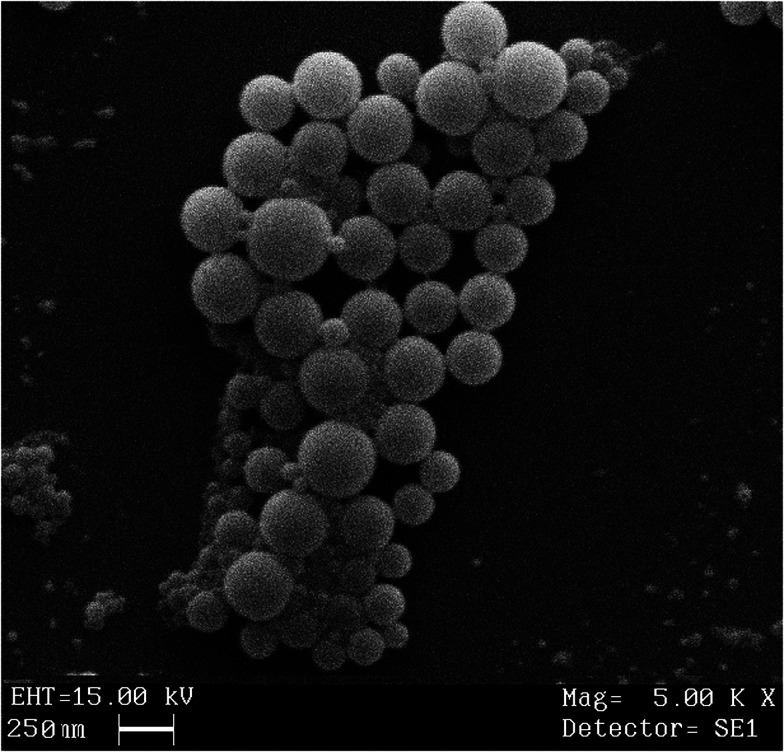
Scanning electron microscopy of optimized formulation.

SEM photomicrographs revealed spherical shape with smooth surface of bromelain loaded nanoparticles (Fig. 5). Microscopic images depicted approximate particle size *i.e.* ∼250 nm which complies with results of photon correlation spectroscopy. Micrographs also disclosed uniform distribution of particles in formulation.

### 2.8 Stability study

The results of stability study of bulk bromelain and optimized formulation stored under accelerated and real-time stability conditions according to ICH guideline are presented in Table 3. Br-NPs showed around 93% drug content expressed on behalf of activity remaining under accelerated storage conditions for six months while bulk bromelain sample showed only 74% drug content. However, under real time storage conditions for 12 months, bulk bromelain powder and L_3_ formulation showed around 76% and 95% drug content respectively. Bromelain followed first order degradation kinetics. The calculated *t*_90_ of bulk bromelain powder and L_3_ formulation at real time stability conditions was 152 and 762 days, respectively (Table 3). Numerical values of *K*_cal_/*t*_90_ suggested that optimized formulations would be able to assure almost two-year shelf life (*t*_90_) for the product whereas bulk bromelain will remain stable for almost five months at room temperature (Table 3). Bulk bromelain powder showed color change from pale buff to light brown. However, no color change was observed in bromelain loaded L_3_ formulation. Thus, results of stability study confirmed significant improvement in stability of bromelain entrapped in Eudragit L 100 nanoparticles compared to bulk bromelain powder.

**Table 3:** Stability data of bulk bromelain and optimized formulation stored under accelerated and real-time stability according to ICH guideline^a^

Storage conditions	Formulation	Bromelain activity remaining (%)						*K*_cal_ (days)^−1^	*t*_90_ (days)
		0 M	1.5 M	3 M	6 M	9 M	12 M		
40 ± 2 °C/75 ± 5% RH	Bulk bromelain powder	100.00 ± 1.78	92.04 ± 2.92	81.96 ± 3.88	74.25 ± 3.60	—	—	1.61 × 10^−3^	65.37
	L_3_	100.00 ± 1.99	97.56 ± 3.89	95.60 ± 3.50	92.71 ± 3.32	—	—	4.61 × 10^−4^	228.78
25 ± 2 °C/60% ± 5% RH	Bulk bromelain powder	100.00 ± 1.78	98.06 ± 2.76	96.17 ± 2.80	88.63 ± 3.35	82.01 ± 2.32	76.19 ± 3.90	6.91 × 10^−4^	152.52
	L_3_	100.00 ± 1.99	99.92 ± 2.48	98.90 ± 3.54	97.26 ± 3.17	96.14 ± 3.23	95.29 ± 2.51	1.38 × 10^−4^	762.62

a Note: values are mean ± standard deviation (*n* = 3). Abbreviations: M, months; *K*_cal_, calculated first order degradation rate constant; *t*_90_, time to reach 90% of initial drug concentration.

### 2.9 Anti-inflammatory activity

**Fig. 6:**
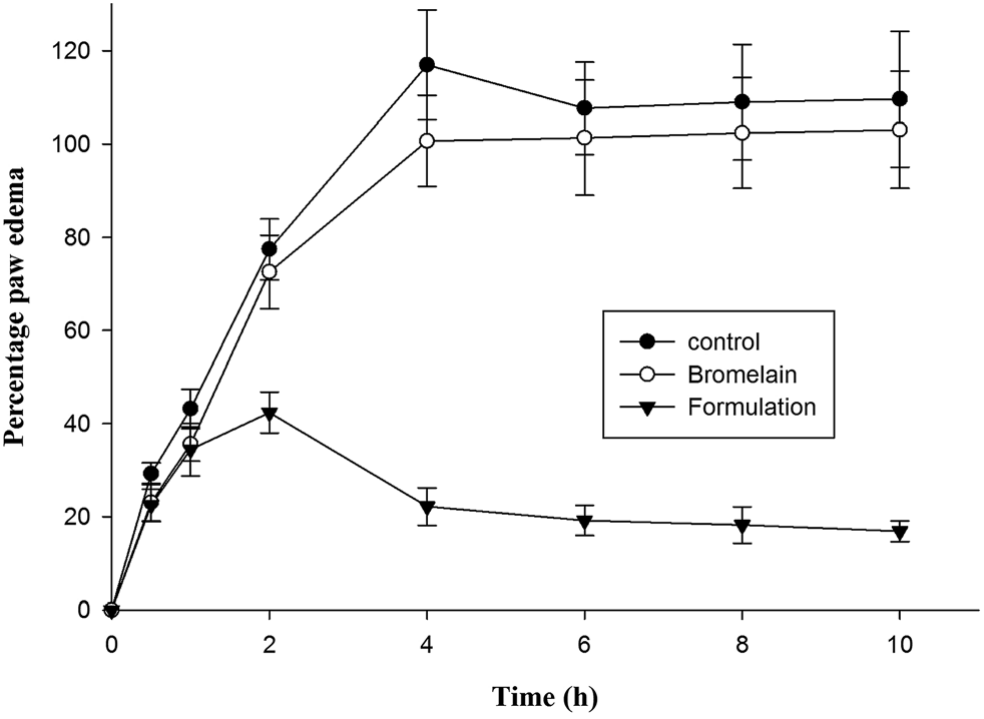
Comparative *in vivo* evaluation of anti-inflammatory activity of bromelain and optimized formulation. Optimized formulation showed statistically significant difference (*p* < 0.001) in percent inflammation at 4 h and later with respect to control as well as bromelain.

Group of animals treated with optimized formulation showed significantly higher *i.e.* 81.02 ± 6.98% reduction in hind paw inflammation in prophylactic treatment after 4 h compared to bromelain solution (13.90 ± 5.67%) (Fig. 6). The percentage reduction in inflammation observed following 10 h of treatment of bromelain solution and optimized nanoparticles was 6.05 ± 1.32% and 84.59 ± 7.02% respectively. According to previous reports available in literature, λ-carrageenan injection induces acute inflammatory changes occurring through cascade of events involving secretion of proinflammatory agents.^48^ During early phase (1–2 h), serotonin, histamine and bradykinins release stimulates fluid extravasation at site of injection while prostaglandins facilitating induction of isoforms of cyclooxygenase plays vital role in late phase of post carrageenan injection.^49^ The outcomes of current study indicated suppression of late phase of carrageenan induced paw edema suggesting bromelain's anti-inflammatory activity may be attributed by inhibition of cyclooxygenase with diminished prostaglandins expression (Fig. 6).^21^ The proteolytic activity of bromelain may also be contributing to its anti-inflammatory potential by inhibiting synthesis of inflammatory mediators, prostaglandin E_2_ and thromboxane A_2_ as well as promoting reabsorption of edema fluids into systemic circulation by enhancing tissue permeability.^15,20,21^ The improved therapeutic potential of formulation might be attributed to encapsulation of bromelain in Eudragit L 100 which prevented bromelain denaturation in gastric environment.^37^ Thus, improved gastric stability and controlled release of bromelain from enteric nanoparticles enhanced its anti-inflammatory activity.

#### 2.9.1 MPO activity

The results of MPO assay showed that carrageenan challenge in control group animals exhibited significantly higher myeloperoxidase activity compared to treatment groups. Pretreatment with bromelain and optimized formulation showed 15.11% and 78.25% reduction in MPO activity respectively compared to saline (Table 4). MPO activity serves as a hallmark of neutrophil accumulation.^50^ Since MPO is an enzyme secreted by neutrophils outside the cells inducing damage to adjacent tissues and contributing to pathogenesis of inflammation. The results confirmed that bromelain ameliorated inflammation by inhibiting trans-endothelial migration and release of MPO facilitating faster apoptosis of neutrophils.^20,21^ Improved therapeutic potential of optimized formulation further confirmed higher protection of bromelain from gastric environment in enteric nanoparticles and availability of higher amount of biologically active bromelain at site of action.

**Table 4:** Effect of bromelain and optimized formulation on MPO activity, NO and cytokines level

S. No.	Groups	Nitrate (nmol g^−1^ tissue)	MPO (U mg^−1^ of protein)	TNFα (pg ml^−1^)	IL-1β (pg ml^−1^)
1	Control	57.14 ± 3.06	11.45 ± 1.94	245.78 ± 45.56	106.23 ± 25.21
2	Bromelain treated	54.01 ± 2.97	9.72 ± 2.08	218.56 ± 41.67	96.11 ± 20.56
3	Optimized formulation treated	32.85 ± 2.05	4.09 ± 1.04	114.34 ± 34.23	51.06 ± 11.09

#### 2.9.2 NO and cytokines (TNFα and IL-1β) estimation

Carrageenan challenge resulted in significant increase in NO synthesis and cytokines (TNFα and IL-1β) level in paw tissues. NO is highly reactive and diffusible radical modulating edema formation by escalating local blood flow and triggering prostaglandins genesis at the spot of inflammation.^51^ Similarly, increased cytokines like TNFα and IL-1β level provokes various inflammatory responses including neutrophil migration in local tissues and induction of acute phase protein synthesis.^22,52^ Therefore, increased NO and cytokines level at site of carrageenan injection provoked inflammation. However, pretreatment of animals with optimized formulation and bromelain guarded against carrageenan induced elevation of NO and cytokines respectively (Table 4). Bromelain's inhibitory effect on release of various inflammatory mediators like TNFα, IL1-β and NO by inhibiting various molecular pathways including NF_KB_ overexpression as well as improving expression of Nrf_2_ pathway has contributed to its anti-inflammatory activity.^50,53–55^ Bromelain nanoparticles produced significantly higher reduction in level of inflammatory modulators compared to bromelain solution due to enhanced gastro-stability of bromelain in enteric formulation. Thus, it can be concluded that usefulness of enteric nano-formulation of bromelain in reduction of cytokine storm is based on enhanced gastro-stability, inhibition of neutrophil infiltration and release of pro-inflammatory cytokines.

## 3. Experimental procedure

### 3.1 Materials

Polymer (Eudragit L100) was procured as a gratis sample from Jubilant life science, India. Potassium dihydrogen phosphate, sodium hydroxide, hydrochloric acid, poly vinyl alcohol, mannitol, cadmium filing, zinc sulphate were acquired from Qualigens fine chemicals, Mumbai, India. Bromelain, casein, tyrosine, carrageenan and trichloro acetic acid (98.0%) was procured from Hi-media laboratory Pvt. Ltd, Mumbai, India. Ethanol, dichloromethane (DCM) and iso-propanol were obtained from Merck, Germany. Hexadecyl trimethylammonium bromide (HTAB) and *o*-dianisidine dihydrochloride was obtained from Sigma Chemicals Co. (St. Louis, MO, EUA). Other reagents and solvents used were of analytical grade. Milli-Q pure water (Millipore Elix water purification system) was used during study.

### 3.2 Preparation of bromelain laden nanoparticles (Br-NPs)

Br-NPs were formulated by double emulsion solvent evaporation method with minor variation of method explained earlier.^32^ Concisely, an aqueous solution of bromelain (15 mg) containing stabilizer (0.1% w/v tween-80) and mannitol (0.25% w/v) was emulsified with organic phase under homogenization speed of 12 500 rpm for 10 min at 4 °C. Organic phase was composed of Eudragit L 100 (75, 150, 225 and 300 mg) dissolved in mixed solvent system of DCM : ethanol : IPA (5 : 6 : 4). The resulting w/o emulsion was further emulsified in aqueous solution of polyvinyl alcohol (1% w/v) containing mannitol (5% w/v) as cryoprotectant using ultrasonic disruptor (PCI analytics 500F) for 5 min at 4 °C in an ice bath. Subsequently, organic solvent was evaporated from multiple emulsion at room temperature under magnetic stirring for 14–16 h. The resulting suspension of nanoparticles was centrifuged at 25 000 rpm for 20 min at 4 °C to collect nanoparticles as pellets. The collected particles were washed thrice with milli-Q water and subjected to lyophilization. Various batches of nanoparticles prepared by varying drug–polymer ratio, volume of external aqueous phase, homogenization speed, type and concentration of stabilizer are presented in Table 1.

### 3.3 Physicochemical characterization of nanoparticles

#### 3.3.1 Proteolytic activity

Modified casein digestion method specified in USP (XXVII) was used to determine the proteolytic potential of bromelain. Briefly, buffered casein substrate (1% w/v, pH 6.0 ± 0.1) was incubated with bromelain solution for 20 min at 37 °C. Subsequently, trichloroacetic acid solution (3 ml, 30% w/v) was added to terminate enzyme–substrate reaction. The precipitated protein was permitted to coagulate for 30 min in an incubator at 37 °C. Thereafter, supernatants collected, filtered and quantified at 280 nm using UV-VIS spectrophotometer (Lab India®,3000^+^, Mumbai, India). Bromelain's proteolytic activity was expressed as casein digestion unit (CDU) defined as microgram of tyrosine formed in 1 min by 1 mg of enzyme under assay condition.

#### 3.3.2 Particle size and zeta potential

Br-NPs were characterized for mean particle size, dispersity index and zeta potential using Nano ZS (Malvern instruments, UK) after dispersing in milli-Q water using an ultra-sonicator (ElmasonicS100H). All reported values were average of three independent samples.

#### 3.3.3 Encapsulation efficiency

Briefly, lyophilized formulation (15 mg) was dissolved in phosphate buffer pH 7.4 (1 ml). Micro BCA protein estimation kit was utilized to determine protein content of samples by microplate reader (Biotek Instruments, Winooski, USA) at 540 nm. Encapsulation efficiency was determined by formula1
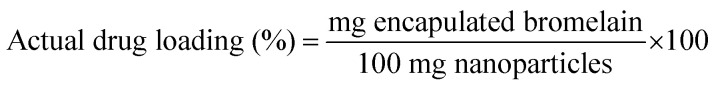
2
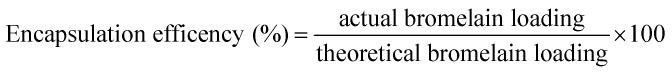


#### 3.3.4 pH dependent stability of nanoparticles

Lyophilized Br-NPs were exposed to different pH conditions like pH 1.2, 5.0, 6.8 and 7.4 corresponding to stomach, duodenum, small intestine and large intestine respectively to predict their stability in GIT fluids. Briefly, nanoparticles equivalent to 10 mg bromelain were incubated into 20 ml of HCl buffer pH 1.2, phosphate buffer pH 5.0, 6.8 and 7.4 in an incubator shaker at 37 ± 0.5 °C and 100 shakes per min for 2 h, respectively. Particle size, surface charge, polydispersity index and percent drug release were determined after incubation of nanoparticles in different buffers respectively.

#### 3.3.5 Dissolution study

Dissolution profile of optimized formulation was determined utilizing USP paddle type dissolution apparatus. Optimized formulation equivalent to 30 mg of bromelain placed in dialysis bags was submerged in pH progressive dissolution medium (250 ml) at 37 ± 2 °C with constant stirring at 100 rpm. Samples were withdrawn at predetermined intervals upto 12 h followed by replacement with equivalent volume of same buffer. The amount of bromelain released was estimated utilizing micro BCA protein estimation kit at 540 nm.

#### 3.3.6 Attenuated total reflection-Fourier transforms infrared spectroscopy (ATR-FTIR)

ATR-FTIR spectra of bromelain, Eudragit L 100 and Br-NPs were recorded in spectral region 4000 to 400 cm^−1^ using Bruker EQUINOX 55 FTIR spectrophotometer. All spectra obtained were corrected by applying advanced ATR correction and percentage transmittance was documented using a nominal resolution of 2 cm^−1^.

#### 3.3.7 Powder X-ray diffractometry (p-XRD)

Diffraction pattern of bromelain, Eudragit L100 and optimized formulation was determined on XPERT-PRO, Pan Analytical, powder X-ray diffractometer (Netherland). Measurements were done at 40 kV and 25 mA tube current using Cu line as radiation source. Samples were scanned at diffraction angle ranging from 3.0 to 45.0° 2*θ* at the scanning rate of 2° min^−1^.

#### 3.3.8 Differential scanning calorimetry (DSC)

Thermal behavior of bromelain, Eudragit L100 and optimized formulation was recorded on DSC-204 F1 Phoenix, NETZESH differential scanning calorimeter (Germany). Samples (3–5 mg) clamped in aluminum pan were heated at 10 °C min^−1^ in nitrogen gas environment (40 ml min^−1^) over a temperature range from 40–250 °C.

#### 3.3.9 Scanning electron microscopy (SEM)

The surface morphology of optimized formulation was evaluated through scanning electron microscope (LEO 435 VP) and images were captured. Samples were mounted on brass stubs using carbon paste and coated with gold and palladium using a high vacuum evaporator (SC7640 Polaron Sputter Coater) under an inert environment.

### 3.4 Stability studies

Stability of bromelain bulk powder and optimized formulation stored in amber colored glass bottles was monitored according to ICH guideline for zone III and IV. Samples were analyzed for stability at accelerated and real-time stability conditions for 6 and 12 months respectively. Samples after thawing and re-dispersing were analyzed for bromelain activity, particle size and zeta potential over a period of stability study.

### 3.5 *In vivo* activity

#### 3.5.1 Animals

Wistar rats (150–180 g) of 4–6 weeks were obtained from Institutional Animal House, Department of Pharmacy, Banasthali Vidyapith, India. All experimental protocols for conducting *in vivo* acute anti-inflammatory studies were sanctioned by the IAEC Banasthali Vidyapith, India. Experimental animals were acclimatized at 25 ± 2 °C and 60 ± 5% RH under a 12 h light/dark cycle for a week with unrestricted access to standard animal diet and water *ad libitum* before initiation of experiment.

#### 3.5.2 Acute anti-inflammatory activity

Wistar rats (150–180 g) were randomly grouped into three groups (*n* = 6). Group I, II and III received normal saline (positive control), bromelain solution (5 mg kg^−1^) and optimized formulation (dose equivalent to 5 mg kg^−1^ bromelain) respectively. All treatments were administered prophylactically 1 h before induction of edema. Edema was induced by injecting 100 μl of 1.0% w/v carrageenan solution in normal saline into subplanar region of right hind paw of all test animals. Subsequent paw volume measurements were made using plethysmometer (M/s Ugo Basile, Italy) by immersing hind paw to hair line to ankle at a time interval of 0, 0.5, 1, 2, 4, 6, 8 and 10 h after injection of inflammation inducer. The change in paw volume was expressed as percentage edema compared to paw volume before carrageenan injection. The percentage edema inhibition was determined using formula

3 % edema inhibition = (*V*_p_C__ − *V*_p_T__)/*V*_p_C__

where *V*_p_C__ and *V*_p_T__ is volume of paw in control group and treated group respectively.

#### 3.5.3 Leukocyte myeloperoxidase (MPO) activity

Paw tissues associated MPO activity was evaluated by method explained by Comalada *et al.*, 2005 with slight modification.^56^ Briefly, paw tissues were homogenized in phosphate buffer (50 mM, pH 6.0) containing 0.5% HTAB under ice cold condition. Homogenates were centrifuged at 15 000 rpm and 4 °C for 25 min. An aliquot (250 μl) withdrawn from supernatant was allowed to react with *o*-dianisidine dihydrochloride (1.6 mM) and hydrogen peroxide (0.1 mM) dissolved in phosphate buffer (50 mM, pH 6.0). Samples were analyzed spectrophotometrically at 460 nm for 2 min in a spectrophotometer. MPO activity was explained as amount of enzyme degrading 1 μmol of peroxide per minute at 37 °C and illustrated in units per milligram of wet tissue.

#### 3.5.4 Determination of nitric oxide (NO) level in paw tissues as oxidative stress parameter

Paw tissues collected after 10 h of carrageenan challenge were immediately homogenized in phosphate buffer saline (pH 7.4) at 4 °C and centrifuged. Supernatants collected and stored at −20 °C prior to assessment of NO level as oxidative stress parameter. NO level in paw tissues was measured by estimating stable end products of nitric oxide oxidation *i.e.* total nitrite/nitrates stated by Sastry *et al.*, 2002.^57^ Briefly, tissue homogenate (100 μl, 10%) was mixed with carbonate buffer (400 μl, 50 mM, pH 9.0) followed by inclusion of little amount of cadmium filing. Test samples were incubated for 1 h with uniform shaking. The reaction was ceased by addition of sodium hydroxide (100 μl, 0.35 M) and zinc sulphate (400 μl, 130 mM) with uniform stirring. Supernatant was collected by centrifuging at 15 000 rpm for 15 min. Aliquots (1 ml) withdrawn from supernatants were gently mixed with Griess reagent (1 ml) and incubated for 10 min at 25 °C. Samples were analyzed spectrophotometrically at 545 nm against blank.

#### 3.5.5 Measurement of TNFα and IL1-β level in paw tissues

TNFα and IL1-β level in supernatant of paw tissue homogenates of normal saline, bromelain, optimized formulation treated groups was determined by commercially available rat cytokines ELISA KITS for TNFα and IL1-β according to standard protocols as per manufacturer's recommendation. Tissue protein content was estimated by Bradford dye-binding assay.^58^ Cytokine levels of tissue samples were expressed as pg/mg of total protein of respected sample.

## 4. Conclusion

A variety of factors like stress, trauma, infection, obesity, insect stings, excessive exercise *etc.* triggers inflammation associated with pain in human beings on a daily basis. NSAIDS commonly utilized to relieve inflammation are often associated with side effects. Thus, investigating new drugs with prospective benefits in inflammation along with shuffling through already known drugs is the demand of the hour. Bromelain, a natural source derived bioactive cysteine protease could be put to good use by delivering it as enteric formulation. The results of present study endorsed the hypothesis that enteric formulation could improve the GIT stability and functional outcomes of bromelain in inflammation. Thus, it can be concluded that bromelain loaded enteric nanoparticles can be considered as promising drug delivery system for improving therapeutic efficacy of bromelain *via* oral route. However, scalability, safety and efficacy of formulation needs to be studied in clinics before its therapeutic use.

## Ethical statement

Animal studies were performed in strict accordance with guidelines established for the care and use of laboratory animals by CPCSEA, Ministry of Social Justice and Empowerment, Government of India. The protocol was approved by the Institutional Animal Ethical Committee of Banasthali Vidyapith, Rajasthan, India.

## Conflicts of interest

The authors report no conflicts of interest in this work.

## Supplementary Material
